# Genome-wide identification and analyses of cotton high-affinity nitrate transporter 2 family genes and their responses to stress

**DOI:** 10.3389/fpls.2023.1170048

**Published:** 2023-04-05

**Authors:** Yuanchun Pu, Peilin Wang, Mubashir Abbas, Muhammad Aamir Khan, Jiangling Xu, Yejun Yang, Ting Zhou, Kai Zheng, Quanjia Chen, Guoqing Sun

**Affiliations:** ^1^ College of Agronomy, Xinjiang Agricultural University, Urumqi, China; ^2^ Biotechnology Research Institute, Chinese Academy of Agricultural Sciences, Beijing, China; ^3^ College of Agronomy, Shanxi Agricultural University, Jinzhong, China

**Keywords:** nitrate transporters, bio-information analysis, expression model, stress, cotton

## Abstract

Nitrate transporters (NRTs) are crucial for the uptake, use, and storage of nitrogen by plants. In this study, 42 members of the *GhNRT2* (Nitrate Transporter 2 family) were found in the four different cotton species. The conserved domains, phylogenetic relationships, physicochemical properties, subcellular localization, conserved motifs, gene structure, *cis*-acting elements, and promoter region expression patterns of these 42 members were analyzed. The findings confirmed that members of the NRT2 family behaved typically, and subcellular localization tests confirmed that they were hydrophobic proteins that were mostly located on the cytoplasmic membrane. The NRT2 family of genes with *A.thaliana* and rice underwent phylogenetic analysis, and the results revealed that *GhNRT2* could be divided into three groups. The same taxa also shared similar gene structure and motif distribution. The composition of *cis*-acting elements suggests that most of the expression of *GhNRT2* may be related to plant hormones, abiotic stress, and photoreactions. The *GhNRT2* gene was highly expressed, mainly in roots. Drought, salt, and extreme temperature stress showed that *GhNRT2* gene expression was significantly up-regulated or down-regulated, indicating that it may be involved in the stress response of cotton. In general, the genes of the NRT2 family of cotton were comprehensively analyzed, and their potential nitrogen uptake and utilization functions in cotton were preliminarily predicted. Additionally, we provide an experimental basis for the adverse stress conditions in which they may function.

## 1 Introduction

Nitrogen (N), mainly in the form of nitrate, is a key factor in plant development, affecting various biological processes in plants, such as photosynthesis and protein synthesis (Frink et al., 1999; Evans and Clarke, 2019). Nitrogen fertilizer use has made a significant contribution to agricultural output and global food security, but this use is also accompanied by substantial environmental consequences such as soils acidification and water eutrophication (Guo et al., 2010; Zhang et al., 2015). With the expansion of planting area and the nutritional needs of crops, nitrogen fertilizer use has increased. However, the absorption efficiency of nitrogen is often less than 40%, and the residual nitrogen left in the field can result in serious environmental pollution (Liu et al., 2022). Therefore, it is particularly important to improve the efficiency of nitrogen fertilizer. To gain maximum yields, an enhancement of the nitrogen use efficiency (NUE) of the crop with low nitrogen inputs is necessary (Liu et al., 2021). Plants mainly absorb nitrogen from soil, with nitrate and ammonium nitrogen being the most effective forms for plant nitrogen; these forms of nitrogen are known as available nitrogen and can be absorbed and used directly by plant roots. Although these two forms of nitrogen have similar nutritional effects on higher plants, there are a few differences in absorption and utilization, and the degree of variation differs with plant species and environmental conditions. Insufficient nitrogen supplies can cause changes in the external morphology and internal metabolism of the plant (Wen et al., 2020).

A study found that there were four gene families of ${\text{NO}}_{3}^{\;-}$ transporters involved in ${\text{NO}}_{3}^{\;-}$ absorption, transportation, and storage: NPF/NRT1 (Nitrate transporter 1(NRT1)/Peptide Transporter (PTR) family (NPF), ${\text{NO}}_{3}^{\;-}$ Transporters protein 1/Peptide family), NRT2 (Nitrate transporter 2 family, ${\text{NO}}_{3}^{\;-}$ Transporters protein 2), CLC (Chloride Channel), and SLAC/SLAH (Slowly Activating Anion Channel) (Taochy et al., 2015). The nitrate uptake process in plants is carried out by NRT2 related proteins, which are typical high-affinity nitrate transporters in the plant (Du et al., 2022). The larger rice genome only possesses four NRT2 genes and two NAR2 genes, compared to seven NRT2 genes and two NAR2 genes in the *Arabidopsis* genome (Wang et al., 2018). Even though all of the NRT2 family genes are involved in the transport of ${\text{NO}}_{3}^{\;-}$ , their roles and transcriptional patterns vary. For example, *AtNRT2.1* was found to be mainly expressed in the cortex and epidermis of the roots (Guan et al., 2021). *AtNRT2.3* and *AtNRT2.6* were mainly expressed in roots and young leaves (Dechorgnat et al., 2012), while *AtNRT2.4* was mainly expressed in roots, and plays an important role in nutrient absorption and transportation of ${\text{NO}}_{3}^{\;-}$ through phloem (Kiba et al., 2012). The homologous protein *OsNPF2.4* in rice was found to be involved in the acquisition of low-affinity nitrates. *OsNPF2*.*4* mediates not only nitrate acquisition, but also root-to-stem nitrate transport and nitrogen reactivation from source to sink organs (Xia et al., 2015). After prolonged starvation, the expression of *AtNRT2.5* was highly induced, was the main transporter of high affinity nitrate uptake, and played a major role in nitrate acquisition and it’s reuse in nitrogen-deficient plants (Lezhneva et al., 2014). Additionally, *AtNRT2.7* regulates nitrate content and dormancy in seeds. *AtNRT2.7* was shown to be specifically expressed in seeds and was the only NRT2 transporter located in the vacuole membrane, where it was involved in the storage of ${\text{NO}}_{3}^{\;-}$ (Chopin et al., 2007). In addition to its role in various processes related to ${\text{NO}}_{3}^{\;-}$ , overexpression of *NRT2.1* and *NRT 2.2* in wheat resulted in increased yield. Similarly, overexpression of *NRT2.1*, *NRT2.2*, and *NRT2.3a* in rice increased crop yields by 10-20% (He et al., 2015; Chen et al., 2017). Furthermore, NRT2 family proteins were also shown to be involved in responding to drought and salinity stresses (Akbudak et al., 2022).

In the case of insufficient nitrogen concentration, the chlorophyll concentration of the plant decreases, turning the leaves yellow and weakening its photosynthetic capacity, which ultimately leads to a significant loss of crop yield (Wen et al., 2020). The distribution of nitrogen in plants is concentrated in the most active part of life activities, so adequate nitrogen supply greatly affects the growth and development of plants (Kant, 2018; Vidal et al., 2020). Cotton is the most significant fiber and cash crop, and nitrogen deficiency can lead to dwarfing of cotton plants, loss of branches and bolls, yellowing of leaves, and severe premature aging and early maturation. Excessive non-protein nitrogen in the plant body results in overly luxuriant growth, large leaves, long petioles, light green leaves, continuous birth of axillary buds, more bud shedding, late maturation, susceptibility to diseases and insect attack, an increase in cotton rotten bolls, and poorer fiber quality (Shah et al., 2022). Nitrogen has an effect on the growth and development of cotton in characteristics such as plant growth morphology, leaf morphology and physiological characteristics, and effects on dry matter accumulation and yields (Iqbal et al., 2020). Nitrogen fertilizers showed different degrees of effects on cotton fiber strength, length, uniformity, and macron value. The increase of nitrogen use efficiency helps increase the fiber length and uniformity of cotton, but excessive nitrogen fertilizer dosage can lead to significant decreases in fiber length and uniformity. Fiber strength and macron value tended to decrease with the increase of nitrogen application rate (Khan et al., 2017).

Modern breeding programs often aim to achieve high yields with high nitrogen supply, which can lead to the prevalence of low nitrogen use efficiency varieties (Liu et al., 2021). Although nitrogen use efficiency can be improved through field management, genetic improvement to produce varieties with high nitrogen use efficiency represent a more basic strategy. As a family of high-affinity nitrogen transporters, the NRT2 family is essential for the nitrogen utilization in cotton. In this study, we performed bioinformatics analysis of two major cultivars, *G.hirsutum* and *G.barbadense*, as well as their possible parental materials *G.arboreum* and *G.raimondii*. By applying nitrate, we were able to determine the expression pattern and response of the NRT2 family, as well as the nitrogen supply level of *G.hirsutum*. We also used the expression of the NRT2 family under stress conditions to characterize their coping mechanisms. The results presented here represent a foundation for enhancing the ability to use nitrogen by providing evidence and recommendations for further describing the function of the NRT2 family in cotton.

## 2 Materials and methods

### 2.1 Identification of NRT2 genes in cotton

The complete genome sequence data of *G.hirsutum* (HAU), *G.barbadense* (HAU), *G.arboreum* (CRI), and *G.raimondii* (JGI) were downloaded from the CottonFGD (https://cottonfgd.net/(accessed on 1 September 2022)). The protein-conserved domain PF07690 (MFS_1) of NRT2s was downloaded from the PFAM database (http://pfam.xfam.org), and the identified *A.thaliana* NRT2 gene in the TAIR (https://www.arabidopsis.org/) was also downloaded. The *A.thaliana* NRT2s (AT1G08090, AT1G08100, AT5G60780, AT5G60770, AT1G12940, AT3G45060, AT5G14570) sequences were then used to perform a BUPP search in CottonFGD with an e-value set to 1e-10, in order to obtain the protein database of cotton NRT2. The proteins were aligned using HMM and Blastp, and the domain was reconfirmed using the CCD database (http://www.NCBI.nlm.nih.gov/CDD/) on the NCBI website. Proteins that did not meet the conditions were eliminated, and the remaining non-redundant proteins were listed as cotton NRT2 proteins. The physical and chemical parameters of the four cotton NRT2 protein sequences, including molecular weight, isoelectric point (pI), and protein length, were calculated using the ExPAsy online tool (https://web.expasy.org/protparam/) (Wilkins et al., 1999). Subcellular localization prediction was carried out using the 1) UniPort (https://www.uniprot.org/) 2) WoLF PSORT (https://wolfpsort.hgc.jp/) 3) Cell-PLoc 2.0 (http://www.csbio.sjtu.edu.cn/bioinf/Cell-PLoc-2/) for Subcellar localization prediction. online functional tool.

### 2.2 Multi-sequence alignment and phylogenetic analysis

To explore the evolutionary relationship between NRT2 proteins in different species, the identified NRT2 family sequences of rice (*O.sativa*) and tomato (*Solanum lycopersicum*) were downloaded from NCBI (https://www.ncbi.nlm.nih.gov/) (Cai et al., 2008; Akbudak et al., 2022). The Arabidopsis NRT2 sequences and the cotton NRT2 protein sequences were also obtained. Using these sequences, a phylogenetic tree was constructed using the neighbor-joining method in MEGA 7.0, with a bootstrap value of 1000 and other parameters set to the default value. The resulting tree was then decorated using EVOLVIEW (https://www.omicsclass.com/article/671). Multiple sequence alignments of the protein sequences were generated using MUSCLE with default parameters and subsequently trimmed using trimAl (Kumar et al., 2016).

### 2.3 Conservation motif and gene structure analysis of NRT2 gene in cotton

The protein sequences of cotton were downloaded from cottonFGD and calibrated using MUSCLE with default parameters. A phylogenetic tree was then constructed using the adjacency ligation method with 1000 guided replications in MEGA7.0. To search for conservative motifs, the online tool MEME was used with optimization parameters set to a maximum of 10 conserved domains in each gene, a minimum and maximum width of each motif of 6-50 amino acid sequences, and default settings for other parameters. The gene structure of NRT2 in cotton was predicted using the online tool GSDS (http://GSDS.cbi.pku.edu.cn/) (Hu et al., 2015). Finally, TBtools were used to visualize the results of conserved motif analysis, genome annotation, motif information, and the constructed evolutionary tree of cotton.

### 2.4 Repeat and chromosomal localization analysis of NRT2 gene in cotton

The gene annotation file for four cotton types (*G.arboretum, G.raimondii, G.hirsutum*, *G.barbadense*) was downloaded from cottonFGD. The chromosomal distribution information of the NRT2 gene was obtained from the reference cotton database, and the distribution of NRT2 genes on the four cotton chromosomes was visualized using TBtools and plotted on chromosome location images using MapChart (Chen et al., 2020). To detect gene duplication, the CDS sequences of the NRT2 gene were compared, and gene pairs that met the criteria for tandem repeat genes (i.e., two or more genes within 200 kb and not less than 75% similarity) were identified. The synonymous (Ks) and non-synonymous (Ka) substitution ratios were calculated using TBtools for the gene pairs and CDS sequences of *G.hirsutum, G.arboreum*, and *G.raimondii*. The Ka/Ks value was used to assess the selection pressure for each gene pair during evolution, with Ka/Ks ratios of >1, = 1, and<1 indicating positive selection pressure, neutral evolution, and purifying selection pressure, respectively. To analyze the homology relationship of the orthologous NRT2 gene obtained from cotton and other selected species, a homology analysis plot was constructed using the Multicollinearity Scanning Toolkit MCScanX (Wang et al., 2012).

### 2.5 Analysis of *cis*-acting elements in the promoter region of the NRT2 gene in cotton

Using cottonFGD, the genomes of four different cotton species were obtained. The promoter sequence of the cotton NRT2 family was extracted from the 1500bp upstream region of each gene with the start codon (ATG) based on the location of gene on the chromosome. PlantCare (http://bioinformatics.psb.ugent.be/webtools/plantcare/html/) was then used to identify the *cis*-acting regulatory elements in the promoter sequences. The identified elements were visualized using TBtools.

### 2.6 Expression profiles of NRT2 gene in different tissues of cotton

RNA-seq data for the TM-1 cultivar (PRJNA248163) were downloaded from NCBI (https://www.ncbi.nlm.nih.gov/) to observe the expression patterns of the GhNRT2 family genes. The RNA-Seq data for TM-1 includes various growth stages such as root, stem, leaf, torus, bract, sepal, petal, filament, and anther at different stages of development.

### 2.7 NRT2 gene subcellular localization analysis

The open reading frames (ORF) of GhNRT2 family were inserted into the pCAMBIA1302 vector. This construct was introduced into *A.tumefaciens* EHA105 *(pSoup*), which was subsequently transformed into cotton protoplast (Wang et al., 2022). The protoplast was analyzed by confocal microscope (Laser confocal super-resolution microscope LSM980) with bright field and fluorescence imaging. All the primers used for this experiment are shown in **Supplementary Table S1**.

### 2.8 Spatial-temporal expression analysis of plant material and stress treatment

XLZ-36 (a common *G.hirsutum*) plants were grown in a mixture of vermiculite and nutrient rich soil (1:2, w/w) at 28°C/25°C (light/dark) under a 16 hour photoperiod. Plants grown for three weeks were used in the experiments. For stress treatment, seedlings were treated with 300mM NaCl solution as salt, 4% PEG6000 solution as drought, 42°C as heat, and 10°C as cold for five days, and each treatment consisted of three replications. After treatment, leaves were excised, immediately frozen with liquid nitrogen, and stored at -80°C until analysis.

### 2.9 RNA isolation, cDNA synthesis, and qRT−PCR

Total RNA was extracted from the samples using the FastPure Plant Total RNA Isolation Kit (Polysaccharides and Polyphenolics-rich, RC401-01, Vazyme Biotech, Nanjing, China). The RNA was treated with DNase I to remove any DNA contamination. Reverse transcription was performed on 2 μg of total RNA using the HiScript III All-in-one RT SuperMix Perfect kit for qPCR, R333-01 (Vazyme). Quantitative real-time PCR was performed on three biological replicates using the ChamQ Universal SYBR qPCR Master Mix (Q711-02, Vazyme) with the ABI 7500 Real-Time PCR system. The expression levels of the target genes were normalized to the expression levels of cotton histone H3, which is known for its stable expression across different tissues, developmental stages, and environmental conditions (Zhu et al., 2017). The 2^−ΔΔCT^ method (Livak and Schmittgen, 2001) was used to analyze the relative expression levels of the target genes. The primer sequences are provided in **Supplementary Table S2**.

## 3 Results

### 3.1 Identification of NRT2 genes in G.raimondii, G.arboreum, G.barbadense, and G.hirsutum

To identify NRT2 genes in four cotton species (*G.arboreum, G.raimondii, G.barbadense*, and *G.hirsutum*), the protein sequences of NRT2 genes in *A.thaliana* were used as a query in BLASTp. A total of 42 NRT2 domain-containing genes were identified, with 7, 7, 14, and 14 genes found in *G.arboreum, G.raimondii, G.hirsutum*, and *G.barbadense*, respectively. In *G.hirsutum* and *G.barbadense*, seven genes were identified in the A genome and seven in the D genome. The genes were named according to their chromosome locations in cotton, with names such as *GaNRT2.1-2.7* (*G.arboreum*), *GrNRT2.1-2.7* (*G.raimondii*), *GhNRT2.A1-7*, *GhNRT2.D1-7* (*G.hirsutum*), *GbNRT2.A1-7*, and *GbNRT2.D1-7* (*G.barbadense*). The NRT2 gene in *G.arboreum* (AA) and *G.raimondii* (DD) represented half the number of NRT2 genes in *G.hirsutum* and *G.barbadense* (AADD) in two tetraploid cottons, indicating that the NRT2 family remained conserved during evolution. **Table 1** provides details of the gene ID number, coding region (CDS) length, protein length, molecular weight (MW), isoelectric point (pI), and other properties of the identified cotton NRT2 family proteins. The proteins encoded by the 42 genes showed different physicochemical properties, with protein length varying between 420-540 amino acids, molecular size between 45,834.81-59,513.36, and theoretical isoelectric points (pl) between 8.27-9.61. All of the discovered members of the cotton NRT2 family were found to be basic proteins, and the prediction results of subcellular localization indicate that all 42 cotton NRT2 proteins were localized to the membrane.

**Table 1:** Features of the 42 NRT2 genes (GhNRT2s) identified in four cotton species.

Species	Gene ID	Gene name	Protein Length(aa)	CDS Length(bp)	molecular weight(kDa)	PI	Formula	Subcellular Localization Predicted		
								Cell-PLoc 2.0	WoLF PSORT	UniProt
*G.arboreum*	Garb_01G004360	GaNRT2.1	536	1608	58004.37	9.2	C_2667_H_4059_N_679_O_717_S_28_	Cell membrane	membrane	Cell membrane
	Garb_01G004370	GaNRT2.2	530	1590	57405.64	9.2	C_2629_H_4017_N_673_O_714_S_29_	Cell membrane	membrane	Cell membrane
	Garb_05G009260	GaNRT2.3	530	1590	57367.6	8.8	C_2620_H_4019_N_671_O_718_S_30_	Cell membrane	membrane	Cell membrane
	Garb_07G001310	GaNRT2.4	453	1359	48711.41	8.3	C_2244_H_3462_N_548_O_598_S_32_	Cell membrane	membrane	vacuole membrane
	Garb_08G006470	GaNRT2.5	530	1590	57447.82	9.3	C_2627_H_4035_N_673_O_715_S_30_	Cell membrane	membrane	Cell membrane
	Garb_09G018910	GaNRT2.6	506	1518	54784.64	9	C_2500_H_3843_N_641_O_686_S_29_	Cell membrane	membrane	Cell membrane
	Garb_12G018830	GaNRT2.7	506	1518	54632.37	9.2	C_2508_H_3851_N_641_O_684_S_22_	Cell membrane	membrane	Cell membrane
*G. raimondii*	Grai_02G017950	GrNRT2.1	543	1629	59513.36	9.1	C_2740_H_4180_N_696_O_730_S_30_	Cell membrane	membrane	Cell membrane
	Grai_02G017960	GrNRT2.2	536	1608	57938.25	9.2	C_2663_H_4057_N_679_O_718_S_27_	Cell membrane	membrane	Cell membrane
	Grai_05G011150	GrNRT2.3	530	1590	57266.54	9	C_2617_H_4016_N_670_O_715_S_30_	Cell membrane	membrane	Cell membrane
	Grai_07G001320	GrNRT2.4	455	1365	48998.7	8.6	C_2252_H_3475_N_555_O_601_S_33_	Cell membrane	membrane	vacuole membrane
	Grai_08G026260	GrNRT2.5	530	1590	57503.92	9.3	C_2631_H_4043_N_673_O_715_S_30_	Cell membrane	membrane	Cell membrane
	Grai_09G018280	GrNRT2.6	530	1590	57389.68	8.8	C_2619_H_4033_N_669_O_721_S_30_	Cell membrane	membrane	Cell membrane
	Grai_12G018580	GrNRT2.7	506	1518	54646.44	9.2	C_2510_H_3857_N_641_O_683_S_22_	Cell membrane	membrane	Cell membrane
*G.barbadense*	Gbar_A03G003790	GbNRT2.A1	536	1608	58004.37	9.2	C_2667_H_4059_N_679_O_717_S_28_	Cell membrane	membrane	Cell membrane
	Gbar_A03G003800	GbNRT2.A2	530	1590	57382.6	9.2	C_2627_H_4016_N_672_O_715_S_29_	Cell membrane	membrane	Cell membrane
	Gbar_A05G010270	GbNRT2.A3	532	1596	57525.75	8.8	C_2626_H_4029_N_673_O_721_S_30_	Cell membrane	membrane	Cell membrane
	Gbar_A07G023580	GbNRT2.A4	455	1365	48920.61	8.3	C_2254_H_3473_N_549_O_602_S_32_	Cell membrane	membrane	vacuole membrane
	Gbar_A08G021500	GbNRT2.A5	530	1590	57475.87	9.3	C_2629_H_4039_N_673_O_715_S_30_	Cell membrane	membrane	Cell membrane
	Gbar_A09G015880	GbNRT2.A6	455	1365	48920.61	8.3	C_2254_H_3473_N_549_O_602_S_32_	Cell membrane	membrane	Cell membrane
	Gbar_A12G015690	GbNRT2.A7	506	1518	54632.37	9.2	C_2508_H_3851_N_641_O_684_S_22_	Cell membrane	membrane	Cell membrane
	Gbar_D03G014600	GbNRT2.D1	530	1590	57455.74	9.3	C_2633_H_4023_N_675_O_712_S_29_	Cell membrane	membrane	Cell membrane
	Gbar_D03G014610	GbNRT2.D2	536	1608	57938.25	9.2	C_2663_H_4057_N_679_O_718_S_27_	Cell membrane	membrane	Cell membrane
	Gbar_D05G010870	GbNRT2.D3	530	1590	57266.54	9	C_2617_H_4016_N_670_O_715_S_30_	Cell membrane	membrane	Cell membrane
	Gbar_D07G024320	GbNRT2.D4	455	1365	48988.62	8.6	C_2249_H_3469_N_555_O_603_S_33_	Cell membrane	membrane	vacuole membrane
	Gbar_D09G015620	GbNRT2.D6	542	1626	58741.23	8.7	C_2683_H_4119_N_685_O_736_S_31_	Cell membrane	membrane	Cell membrane
	Gbar_D08G022250	GbNRT2.D5	530	1590	57503.92	9.3	C_2631_H_4043_N_673_O_715_S_30_	Cell membrane	membrane	Cell membrane
	Gbar_D12G015710	GbNRT2.D7	437	1311	47092.81	9.6	C_2158_H_3334_N_560_O_583_S_20_	Cell membrane	membrane	Cell membrane
*G.hirsutum*	Ghir_A03G003830	GhNRT2.A1	536	1608	58020.37	9.2	C_2667_H_4059_N_679_O_718_S_28_	Cell membrane	membrane	Cell membrane
	Ghir_A03G003840	GhNRT2.A2	530	1590	57382.6	9.2	C_2627_H_4016_N_672_O_715_S_29_	Cell membrane	membrane	Cell membrane
	Ghir_D12G015860	GhNRT2.A3	420	1260	45834.81	9.1	C_2116_H_3227_N_531_O_552_S_28_	Cell membrane	membrane	Cell membrane
	Ghir_A07G023910	GhNRT2.A4	455	1365	48960.67	8.3	C_2257_H_3477_N_549_O_602_S_32_	Cell membrane	membrane	vacuole membrane
	Ghir_A08G020890	GhNRT2.A5	530	1590	57447.82	9.3	C_2627_H_4035_N_673_O_715_S_30_	Cell membrane	membrane	Cell membrane
	Ghir_A09G015660	GhNRT2.A5	534	1602	58122.3	8.6	C_2649_H_4045_N_689_O_724_S_31_	Cell membrane	membrane	Cell membrane
	Ghir_A12G015590	GhNRT2.A7	506	1518	54632.37	9.2	C_2508_H_3851_N_641_O_684_S_22_	Cell membrane	membrane	Cell membrane
	Ghir_D03G015080	GhNRT2.D1	520	1560	56529.79	9.4	C_2591_H_3968_N_666_O_697_S_29_	Cell membrane	membrane	Cell membrane
	Ghir_D03G015090	GhNRT2.D2	536	1608	57938.25	9.2	C_2663_H_4057_N_679_O_718_S_27_	Cell membrane	membrane	Cell membrane
	Ghir_D05G010610	GhNRT2.D3	530	1590	57252.51	9	C_2616_H_4014_N_670_O_715_S_30_	Cell membrane	membrane	Cell membrane
	Ghir_D07G023970	GhNRT2.D4	455	1365	48970.65	8.6	C_2250_H_3471_N_555_O_601_S_33_	Cell membrane	membrane	vacuole membrane
	Ghir_D08G021680	GhNRT2.D5	514	1542	55540.7	9.1	C_2533_H_3916_N_650_O_694_S_30_	Cell membrane	membrane	Cell membrane
	Ghir_D09G015140	GhNRT2.D6	542	1626	58755.25	8.7	C_2684_H_4121_N_685_O_736_S_31_	Cell membrane	membrane	Cell membrane
	Ghir_A05G010860	GhNRT2.D7	506	1518	54646.44	9.2	C_2510_H_3857_N_641_O_683_S_22_	Cell membrane	membrane	Cell membrane

### 3.2 Conserved motif and gene structure analysis of NRT2 in cotton

**Figure 1:**
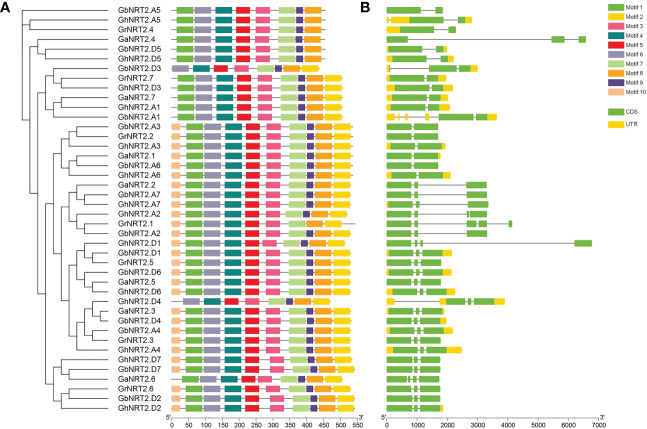
Phylogenetic tree and sequence structures of the candidate NRT2s in four cotton species (*G.arboreum, G.raimondii, G.barbadense*, and *G.hirsutum*). **(A)** Neighbor-joining (NJ) phylogenetic tree of NRT2s in four cotton species. Different colors represent different motifs. **(B)** Gene structures of NRT2s in cotton. CDS are shown as green boxes, UTR are shown as yellow boxes, and the lines between the colored boxes correspond to the introns. Numbers below represent the position.

To investigate the evolutionary relationship between NRT2 members in *G.arboreum, G.raimondii, G.barbadense*, and *G.hirsutum*, a phylogenetic tree was created using 42 cotton NRT2 genes, which were categorized into three subfamilies. The resulting tree showed that NRT2 genes from the four cotton species were closely grouped in each subfamily. In order to study the gene structure of the cotton NRT2 family genes, coding sequences were mapped onto the genome sequences of exon/intron sequences. The analysis revealed that the exon/intron structure of the cotton NRT2 gene was generally well-conserved across different varieties (**Figure 1**).

To gain further insight into the structural characteristics of the NRT2 genes in cotton, we employed the MEME server to identify ten conserved motifs. We found that motifs 2, 3, 4, 5, 6, 7, 8, and 9 were present in all NRT2 protein sequences (**Figure 1**). Groups I and II contained the same motifs (1, 3, 4, 5, 6, 7, 8, 9), whereas *GbNRT2.D3* and *GhNRT2.D4* in Group III lacked motifs 1 and 10. This result suggests that members of the same family have similar functions.

To further examine the phylogenetic relationships, the arrangement of exons and introns of the cotton NRT2 genes were studied. The findings showed that the number of exons and introns in the cotton NRT2 gene ranged from 2 to 5. Group I and II genes shared a similar structure, with one intron and two exons, while most members of Group III had a comparable exon-intron structure and gene length (**Figure 1**).

### 3.3 Chromosomal location, gene duplication and evolutionary relationships of Gossypium NRT2 family genes

**Figure 2:**
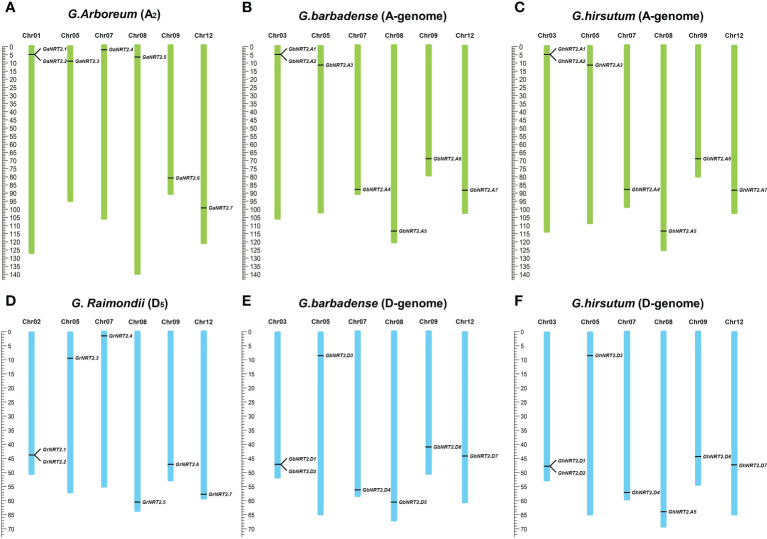
Chromosome distribution and duplication of GhNRT2s. **(A)** 7 *GaNRT2s* were mapped on 6 chromosomes in *G.arboreum*; **(B)** 7 *GbNRT2s* were mapped on 6 chromosomes in *G.barbadense *(A-genome); **(C)** 7 *GhNRT2s* were mapped on 6 chromosomes in *G.hirsutum* (A-genome); **(D)** 7 *GrNRT2s* were mapped on 6 chromosomes in *G.raimondii*; **(E)** 7 *GbNRT2s* were mapped on 6 chromosomes in *G.barbadense* (D-genome); **(F)** 7 *GhNRT2s* were mapped on 6 chromosomes in *G.hirsutum* (D-genome).

Cotton NRT2 genes are not distributed on all 13 chromosomes. Based on the annotation of four cotton genomes, 14 *GhNRT2s* and 14 *GbNRT2s* genes were assigned to 12 chromosomes, of which 7 were located on the A-genome, 2 genes on chromosome A03 (*GbNRT2A1, GbNRT2A2* and *GhNRT2.A1, GhNRT2.A2*), and one gene on chromosome A05, 7, 8, 9, 12 (*GbNRT2.A3, GbNRT2.A4, GbNRT2.A5, GbNRT2.A6, GbNRT2.A7* and *GhNRT2.A3*, *GhNRT2.A4*, *GhNRT2.A5, GhNRT2.A6, GhNRT2.A7*). The remaining 7 were located on the D-genome, 2 genes on chromosome D03 (*GbNRT2.D1, GbNRT2.D2* and *GhNRT2.D1, GhNRT2.D2*), D05, 7, 8, 9, and a gene on chromosome 12 (*GbNRT2.D3, GbNRT2.D4, GbNRT2.D5, GbNRT2.D6*, *GbNRT2.D7* and *GhNRT2.D3, GhNRT2.D4, GhNRT2.D5, GhNRT2.D6, GhNRT2.D7*). The NRT2 gene of *G.arboreum* was assigned to chromosomes 1 (*GaNRT2.1 and GaNRT2.2*), 5 (*GaNRT2.3*), 7 (*GaNRT2.4*), 8 (*GaNRT2.5*), 9 (*GaNRT2.6*), and 12 (*GaNRT2.7*). A total of 7 genes of *G.raimondii* were assigned to two genes on chromosome 02 (*GrNRT2.1* and *GrNRT2.2*) and one gene on 05 (*GrNRT2.3*). There was one gene each on chromosomes 7 (*GrNRT2.4*), 8 (*GrNRT2.5*), 9 (*GrNRT2.6*), and 12 (*GrNRT2.7*) (**Figure 2**). Previous studies have shown that *G.arboreum* and *G.raimondii* were donor species for the A and D genomes.

The number of *GhNRT2* and *GbNRT2* genes present in the A and D genome of cotton was consistent with the number of *GaNRT2* and *GrNRT2* genes. Tandem repeats of *NRT2.1* and *2.2* were observed in all four types of cotton, indicating possible gene duplication. To gain insight into the relationship between NRT2 genes and potential gene duplication within the cotton genome, we analyzed the occurrence of tandem duplication and segmental duplication during the evolution of this gene family. We calculated the non-synonymous differentiation level (Ka) and the synonymous differentiation degree of 35 homology pairs to infer possible selection pressure. The Ka/Ks ratio was used to assess the selection pressure on repeating genes. A Ka/Ks ratio of 1 suggests pseudo genes with natural selection; a Ka/Ks ratio<1 indicates that duplicate genes had a tendency to purify, and a Ka/Ks ratio >1 indicates accelerated evolution with positive selection. Our analysis revealed that the Ka/Ks ratio of all 25 gene pairs was less than 0.5, leading us to hypothesize that the NRT2 gene family in cotton experienced strong pressure for purification selection.

**Figure 3:**
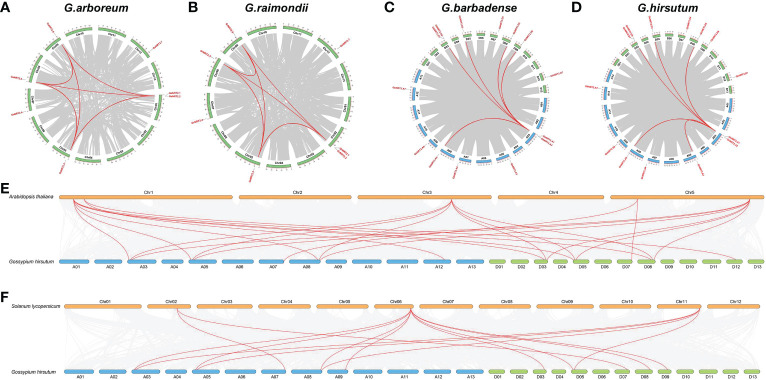
Synteny analysis of the GhNRT2s genes. **(A)** *G.arboreum*; **(B)** *G.raimondii*; **(C)** *G.barbadense*; **(D)** *G.hirsutum*; **(E)** *Arabidopsis thaliana* and *G.hirsutum*; **(F)** *Solanum lycopersicum L.* and *G.hirsutum*.

To further investigate the genetic origins and evolutionary relationships of *NRT2s* in cotton, we conducted collinear analyses of cotton in comparison with *A.thaliana*, rice, and tomato. The collinear analysis results of four cotton varieties were mainly divided into two forms within species. In *G. arboreum* and *G. raimondii*, *NRT2.1* and *NRT2.2* were collinear with *NRT2.3, NRT2.5*, and *NRT2.6*, while *NRT2.3* was collinear with *NRT2.5* and *NRT2.6.* In *G. barbadense* and *G. hirsutum*, *NRT2.A1/A2* was collinear with *NRT2.A5, NRT2.D1/D2, NRT2.D3, NRT2.D4, NRT2.D5*, and *NRT2.D6* (**Figure 3**). These results suggest that the internal gene sequencing of the homologous fragment was conserved and may also be functionally conserved. Additionally, we compared different species and found that *AtNRT2.1*, *AtNRT2.4*, and *AtNRT2.6* were collinear with *GhNRT2.1, GhNRT2.3*, and *GhNRT2.5* in *G. hirsutum*, while *AtNRT2.5* was collinear with *GhNRT2.6* and *GhNRT2.7*, and *AtNRT2.7* was collinear with *GhNRT2.4* (**Figure 3**). These results indicate that these genes possess high similarities in structure and function across species.

### 3.4 Multiple sequence alignment and phylogenetic analysis

In order to study the evolutionary relationship between cotton NRT2 genes (*G.arboreum, G.raimondii, G.barbadense* and *G.hirsutum*) and other species, we identified 57 NRT2 genes in 7 genomes (cotton, Arabidopsis, rice, tomato), including 14 each for *G.barbadense* and *G.hirsutum*, 7 each for *G.arboreum* and *G.raimondii*, 4 for rice, 4 for tomatoes, and 7 for Arabidopsis. We then performed phylogenetic analysis of these 57 NRT2 protein sequences, and constructed an evolutionary tree using the Neighbor-Joining (NJ) method. Phylogenetic analysis revealed the relatively deep evolutionary origins of these genes as well as recent duplications.

**Figure 4:**
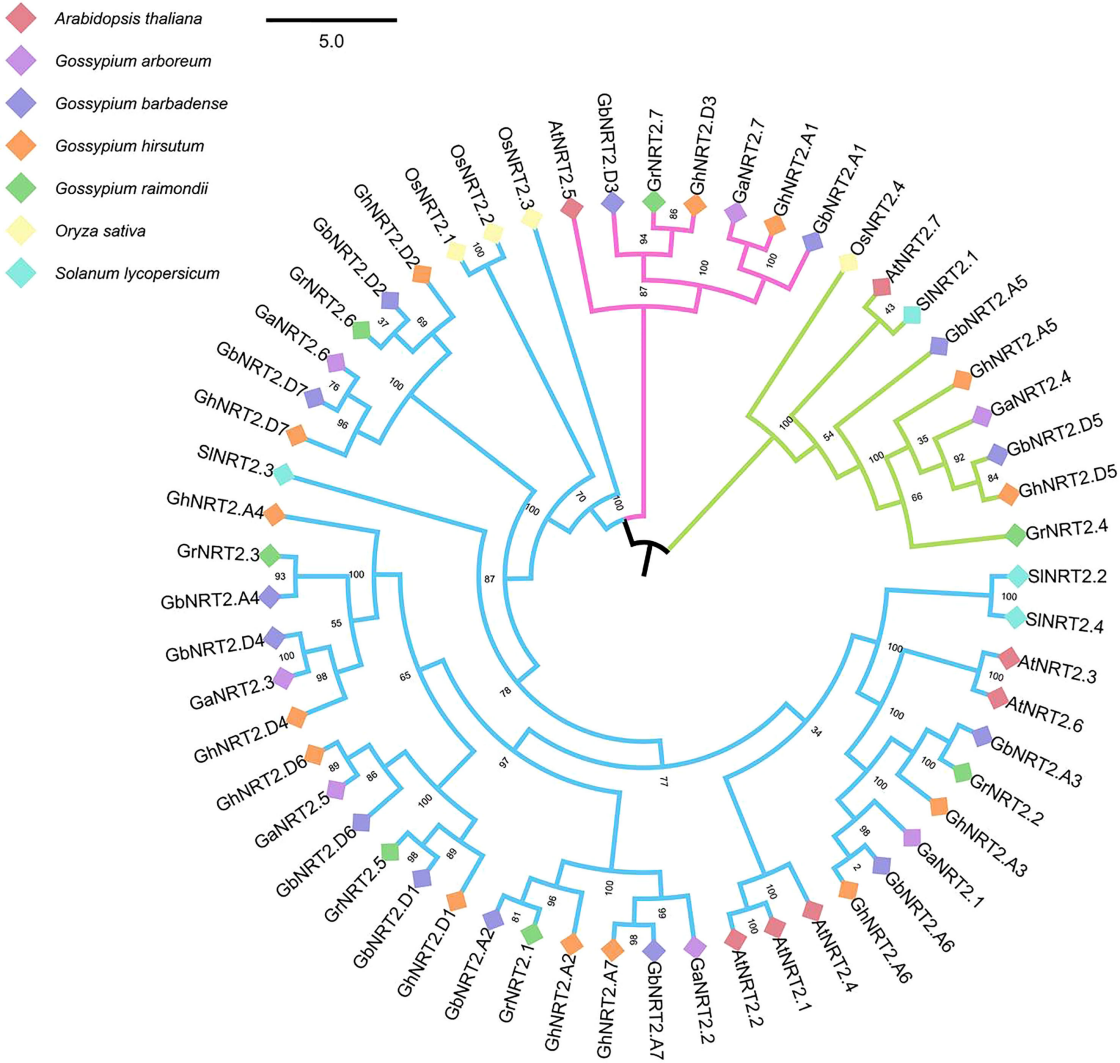
Phylogenetic analysis of NRT2 gene families Phylogenetic analysis of the NRT2 gene families in cotton, Arabidopsis, rice, and tomato. Different groups are colored by a special line. Different plants use small squares of different colors. The number beside the branch indicates the bootstrap value of each group from 1,000 replicates.

According to the topology and boot values of the tree, the candidates were divided into three groups: group I–III and group I had 7 members, including Arabidopsis *AtNRT2.5, 2 GhNRT2s, 2 GbNRT2s, GrNRT2.7* and *GaNRT2.7*; Group II had 9 members (*OsNRT2.4, AtNRT2.7, SINNRT2.1, 2 GbNRT2s, 2 GhNRT2s* and *GaNRT2.4*); Group III was the largest and included 3 *SINNRT2s*, 5 *AtNRT2s*, 3 *OsNRT2s*, 10 *GbNRT2s*, 10 *GhNRT2s*, 5 *GrNRT2s*, and 5 *GaNRT2s* (**Figure 4**). The results showed that the genes located in group I were functionally similar to *AtNRT2.5* of *A.thaliana*, and played a role in nitrate acquisition and reuse in nitrogen-deficient plants (Chopin et al., 2007). The related genes located in group II were consistent with *AtNRT2.5* of *A.thaliana* and *OsNRT2.4* of rice. Acquisition and root-to-stem nitrate transport had the same function (Lezhneva et al., 2014; Xia et al., 2015).

### 3.5 *Cis*-element analysis in the promoter regions of cotton NRT2 genes

To understand the underlying regulatory mechanism, we predicted *cis*-acting elements and analyzed the 1,500bp upstream promoter region of 42 cotton NRT2s *via* PlantCARE. In addition to some common basic core elements (e.g., A-box, TATA-box) and photo-responsive elements (e.g., ACE), the remaining elements were divided into five broad categories, including hormone-responsive elements, protein-binding sites, special response elements, tissue-specific expression elements, and biotic and abiotic response elements. Hormonal response elements included abscisic acid (ABRE), salicylic acid (TCA-element), gibberellin (P-box, TATC-box), jasmonic acid (TGACG motif, CGTCA motif), and auxin (AuxRR-core). In the group of protein binding sites, 13 *cis*-acting elements were found to interact specifically with the C-terminal domain of 60K, and in the group of special reaction elements, 4 cotton *NRT2s* may be involved in the regulation of zein metabolism (O2 site). In the tissue-specific expression element group, 21 cotton NRT2s may be associated with meristem activation and expression (GCN4_motif, HD-Zip 1, and CAT-box). In the biotic and abiotic *cis*-responsive *cis*-component group, 3, 12, and 17 cotton NRT2s may be involved in hypoxia (GC-motif), low temperature (LTR), and defense and stress (TC-rich repeats) response processes, and 23 cotton NRT2s were associated with sites in drought (MBS) response.

### 3.6 NRT2 gene subcellular localization analysis

**Figure 5:**
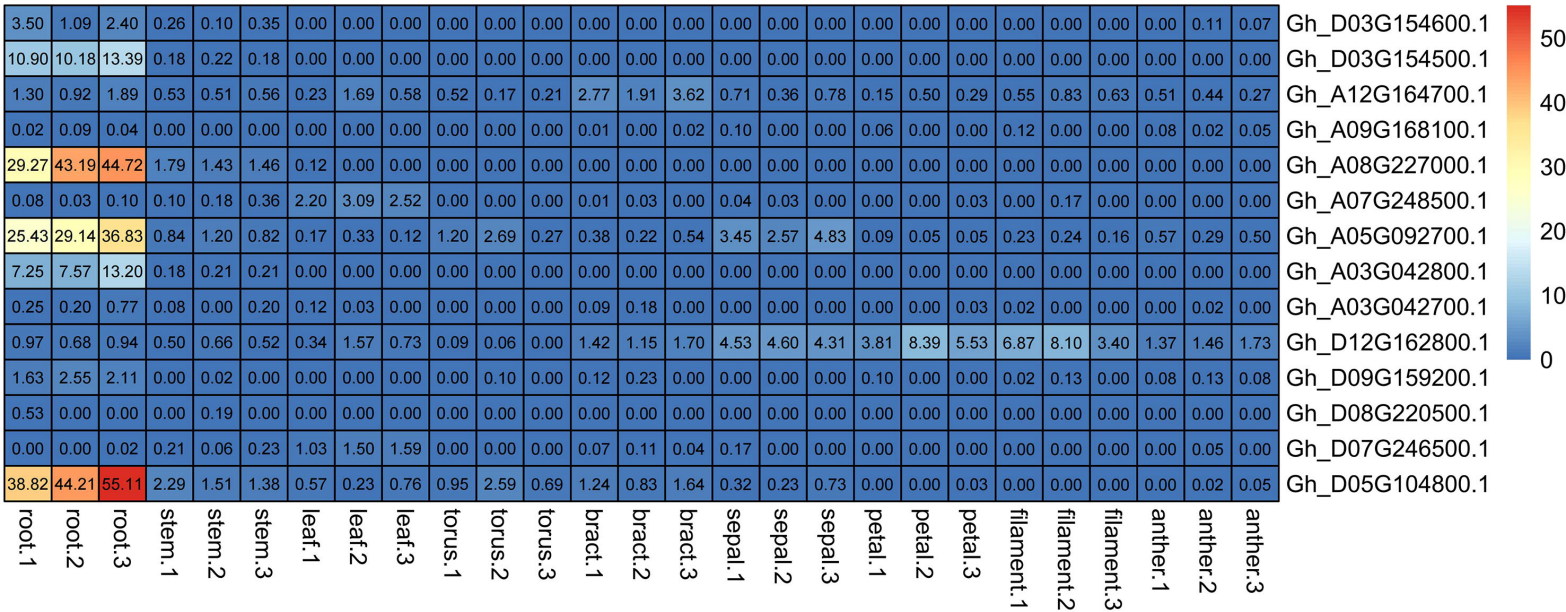
*GhNRT2s* genes expression in different organ. Expression heat map of GhNRT2s family genes in different organ transcriptomes. All data were downloaded in cottonFGD (https://cottonfgd.net/, on October 18, 2022).

Three *G.hirsutum* genes were arbitrarily chosen for subcellular localization in order to assess the accuracy of the software’s predictions. The pCAMBIA1302-*GhNRT2.A1*, *GhNRT2.A3*, and *GhNRT2.A5* vectors were constructed and transformed into the *Agrobacterium tumefaciens* strain EHA105 (*pSoup*) to determine the localization of the GhNRT2.A1, GhNRT2.A3, GhNRT2.A5 proteins by transient expression in cotton protoplast. The GhNRT2.A1, GhNRT2.A3, and GhNRT2.A5 proteins appeared to be localized to the cell membrane, according to the GFP fluorescence signal generated by laser confocal microscopy (**Figure 5**).

### 3.7 Analysis of NRT2 gene expression in different tissues of cotton

**Figure 6:**
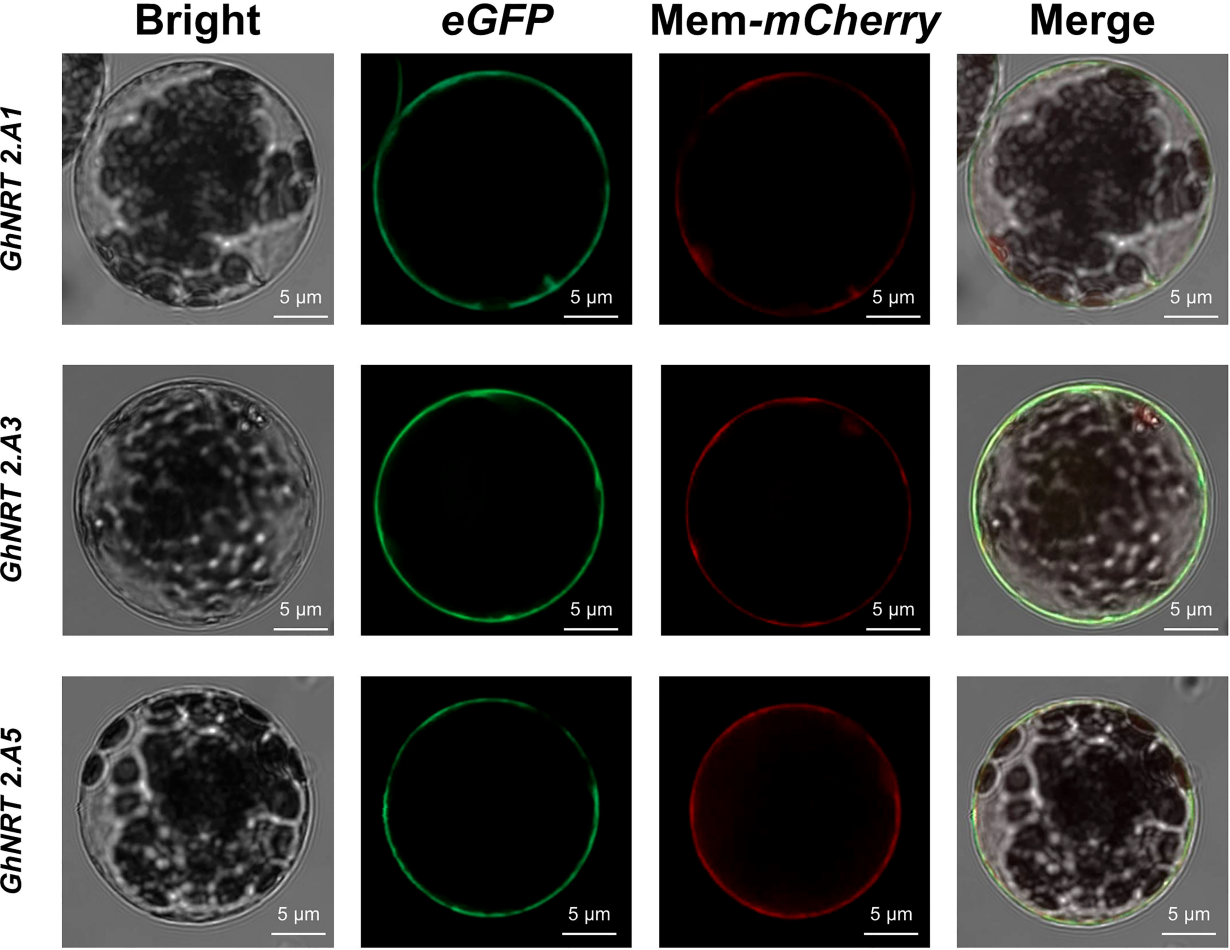
*GhNRT2s* genes Subcellular localization. Subcellular localization assay with cotton protoplast using the pCaMV35S::*GhNRT2.A1*/*GhNRT2.A3/GhNRT2.A5*-eGFP construct. Red fluorescence is mCherry of membrane localization. Scale bars = 5 µm.

**Figure 7:**
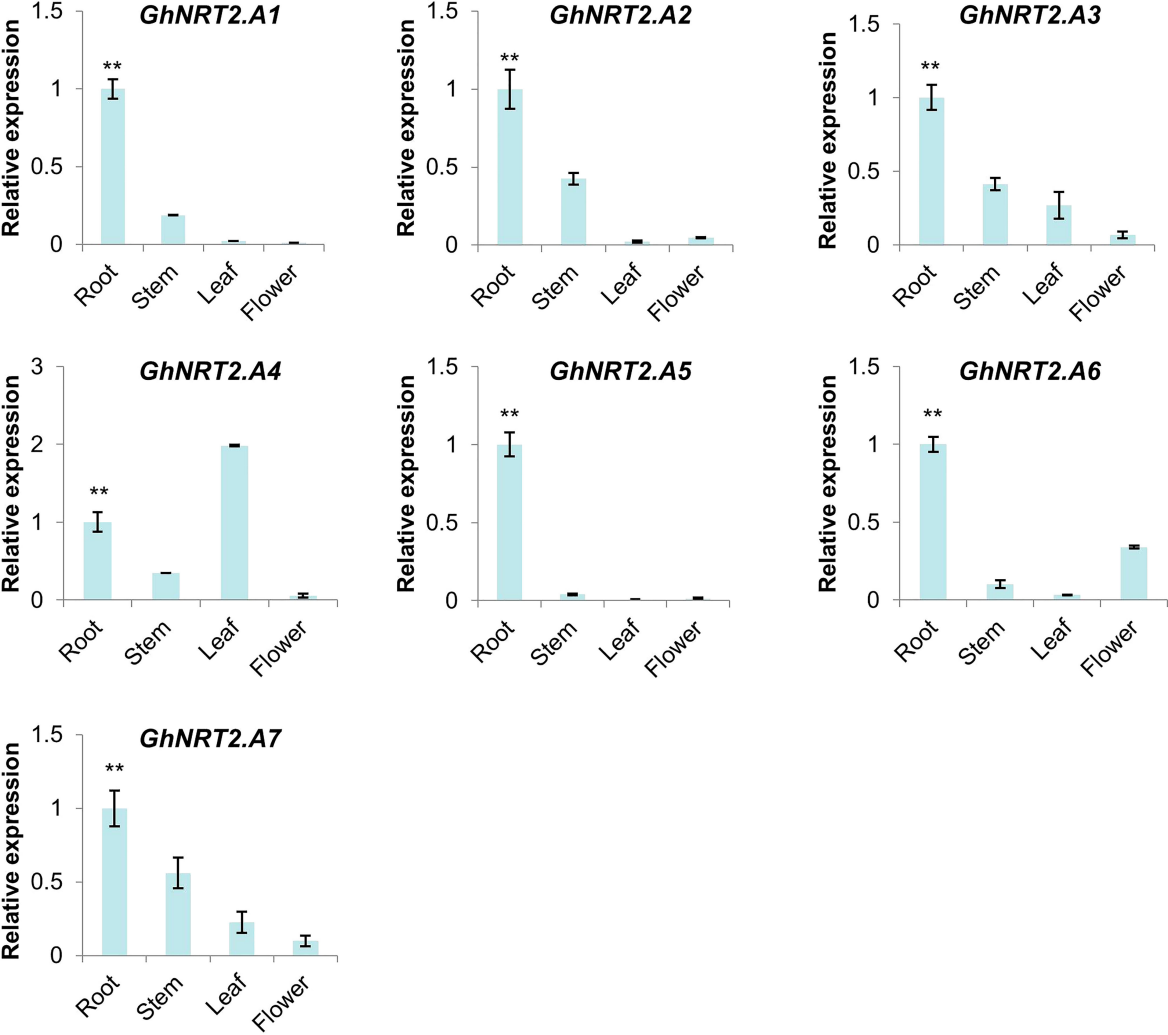
The expression of *GhNRT2.A1-A7* in different organ. The qRT-PCR result of *GhNRT2.A1-A7* in root, stem, leaf, and flower of *G.hirsutum*. Values represent the mean ± S.D (n = 3 replicates). **p< 0.01, Dunn’s test.

RNA-seq expression data in different tissues of the cotton NRT2 gene family were obtained from the cotton database CottonFGD. Due to the lack of data, there was only relevant data for *G.hirsutum* in the database, so we chose *G.hirsutum* for characterization. From the RNA-seq data, the NRT gene family showed obvious root specificity. Almost all of the NRT genes had high expression specifically in the roots. In addition to this, the NRT genes were also expressed in the leaves and stems. Among GhNRT2 genes, *GhNRT2.A4* showed specific high expression in leaves, while *GhNRT2.A6* and *GhNRT2.D7* genes were expressed as a specificity of flower organs (**Figure 6**). To verify the accuracy of these data, we verified this result by qRT-PCR. Considering the homologous relationship, we chose *GhNRT2.A1-A7* to determine their expression level (**Figure 7**). The results were almost identical to the RNA-seq data, which showed expression at specific high levels in the root and in both leaves and stems (**Figure S1**). Among GhNRT2 genes, *GhNRT2.A4* showed high expression in leaves, and these results were consistent with the correlation between the expression pattern of these genes and their driving function.

### 3.8 Analysis of NRT2 gene expression in different stress of cotton

**Figure 8:**
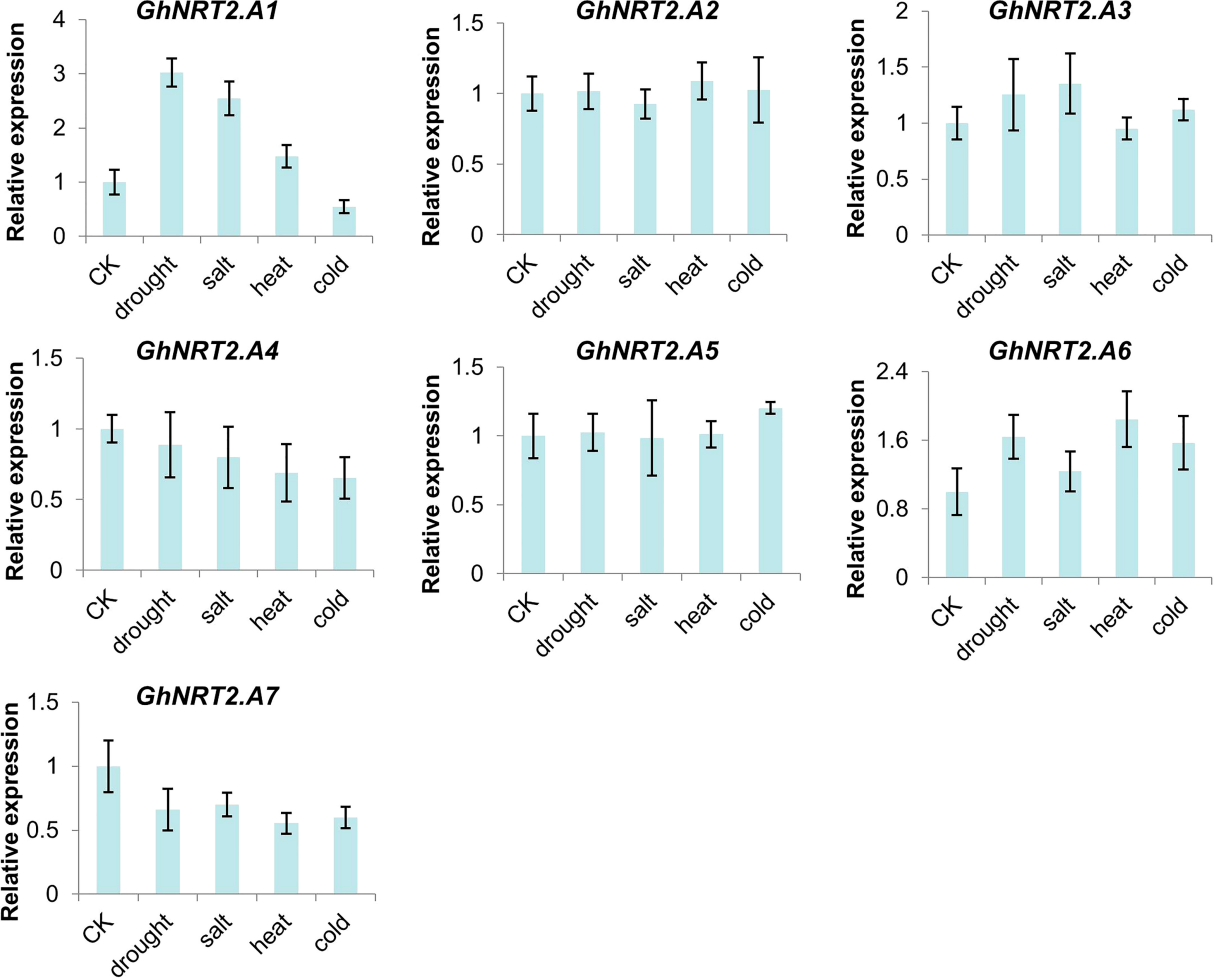
The expression of *GhNRT2.A1-A7* in different treatments. The qRT-PCR result of *GhNRT2.A1-A7* in root of *G.hirsutum* treated with drought (4% PEG6000), salt (200 mM NaCl), heat (42°C), and cold (10°C). Values represent the mean ± S.D (n = 3 replicates).

To ascertain the response of the NRT2 family genes in cotton to salt, drought, and temperature stress, salt and drought processes by NaCl were simulated. Then, using PEG6000 to imitate drought and salt processes at a high temperature of 42°C and a low temperature of 10°C, expression analysis was carried out. The results showed that the expression levels of *GhNRT2.A1-A7* responded to stress, among which *GhNRT2.A1*, *GhNRT2.A3*, and *GhNRT2.A6* were up-regulated under stress condition, and the expression of *GhNRT2.A4* and *GhNRT2.A7* was down-regulated. Expression levels of *GhNRT2.A2* and *GhNRT2.A5* did not change significantly (**Figure 8**). The results were almost identical to the RNA-seq data (**Figure S2**).

## 4 Discussion

Nitrate is an essential component for plant growth since it is both a significant nutrient in the nitrogen absorption route and an important signal for plant development. Crop yield is directly related to the availability of nitrogen; in the event of its deficiency, a plant’s chlorophyll concentration level declines, which results in poor photosynthesis activity (Zhao et al., 2022).

In cotton, we found that the *NRT2* gene in *G.arboreum* (AA) and *G.raimondii* (DD) were essentially half of the total *G.hirsutum* and *G.barbadense* (AADD) *NRT2* genes in two tetraploid cottons. We showed that there was no copy number variation in the NRT gene during doubling from diploid to tetraploid. Additionally, based on where these genes were located on the genome, they remained almost identical to their diploid parents, which indicates that the NRT2 family remained conserved during the process of evolution. The NRT2 gene family in cotton has experienced strong purification selection pressure.

*Cis*-acting regulatory element analysis of the cotton NRT2 promoter regions revealed that it has an abundant number of hormone-responsive elements, protein-binding sites, special response elements, tissue-specific expression elements, and biotic and abiotic response elements. Different hormonal response elements like abscisic acid, salicylic acid, gibberellin (P-box, TATC-box), jasmonic acid, and auxin were also part of the ***cis***-acting regulatory elements. Interestingly, although almost all genes in the GhNRT family are specifically expressed in the roots, most GhNRTs still have photoresponsive factors in the promoters of the genes, indicating that the expression of most GhNRTs is also regulated by light. The latest research proves that ET (ethylene)/JA (jasmonic acid)-NRT signaling module participates in ${\text{NO}}_{3}^{\;-}$ _−_ distribution between plant roots and aerial parts in a nitrate reductase-dependent manner, regulating stress tolerance and plant growth (Zhang et al., 2014)

Under stress conditions, the cotton *NRT2* gene family responded differently i.e., *GhNRT2.A1, GhNRT2.A3* and *GhNRT2.A6* were up-regulated under stress conditions, while the expression of *GhNRT2.A4* and *GhNRT2.A7* were down-regulated. Although we have not performed expression or knockout experiments to demonstrate that genes from the GhNRT family increase stress tolerance in cotton, changes in the expression of these genes in response to stress suggest that they may be involved in the stress response. Zhao and colleagues have demonstrated that the plant may have a Cl/NO**_3_** cooperative transport system through the control of NRTs, which is crucial for salt resistance, especially for Cl sensitive plants (Zhao et al., 2022). In a previous report, the expression of *OsNRT2.3* in rice was shown to increase during high temperature stress, increasing high temperature tolerance and nitrogen uptake levels (Zhang et al., 2022), In tomato (*Solanum lycopersicum* L.), the SlNRT2 family proteins were also involved in the response to drought and salinity (Akbudak et al., 2022). The responses of the *SlNRT2* genes to drought and salinity stresses were diverse, and they were neither stress- nor tissue-specific (Akbudak et al., 2022). In wheat (*Triticum aestivum* L.), the TaNRT2 gene family improved high salt tolerance (Shi et al., 2022).

The expression levels of the NRT2 family of genes changed by various degrees as a result of our four adversity treatments. *GhNRT2.A1* and *GhNRT2.A7* in particular demonstrated consistent up- or down-regulation of the two genes under various stresses. This implies that dealing with various adversities may involve the same biological pathways. Additionally, because there was only a single sample time in this investigation, it would be possible to more accurately characterize the pattern of gene expression in the *NRT2* family in response to stress. These mechanisms still need to be investigated further. The genetic resources available for enhancing cotton resistance to environmental stresses could be greatly increased by focusing on specific *NRT2* members that coordinate nitrate absorption and transport in response to signals from various stress response pathways.

Based on our findings, we suggest that *NRT2* transporters could be considered as potential targets for plant adaption to varied forms of nitrogen supply and for improving NUE. Since previous studies have shown that the *NRT2* family has a role in responding to drought and waterlogging stresses, this same functionality should also be investigated for cotton. Thorough study of the gray leaf spot and bacterial wilt RNA-seq data confirmed that the expression of the NRT1/PTR subfamily members was down-regulated after pathogen attack (Xia et al., 2022). Reports are also available which imply that NRT2 family proteins play a role in plant response to light, which ultimately increases photosynthesis. Previous studies on *A.thaliana* revealed that *AtNRT2.1* was up-regulated during the day and down-regulated at night, which might be hindered by sucrose (Lejay et al., 1999). Our next step is to investigate introgression or expressing altered forms of *NRT2* with higher uptake or sensing activity in local cultivars, which may optimize NUE and other genetic traits, and hence can increase the crop yield with minimized fertilizer application.

## Data availability statement

The datasets presented in this study can be found in online repositories. The names of the repository/repositories and accession number(s) can be found in the article/**Supplementary Material**.

## Author contributions

Conceptualization, YP and PW. Methodology, YP. Software, YP. Validation, PW. Formal analysis, JX. Investigation, TZ and YY. Resources, KZ and GS. Data curation, MK. Writing original draft preparation, YP and PW. Writing review and editing, PW and MA. Visualization, QC. Supervision, GS. Project administration, GS. Funding acquisition, GS. All authors contributed to the article and approved the submitted version.

## Supplementary material

Supplementary Figure 1 The expression of *GhNRT2.A1-A7* in different organs compared by RNA-seq data and RT-PCR data (For easy comparison, all root data were set to 1).

Supplementary Figure 2 The expression of *GhNRT2.A1-A7* in different treatments compared by RNA-seq data and RT-PCR data (For easy comparison, all CD data were set to 1).
