# Supplementary figures and images for: Genome-wide identification and analyses of cotton high-affinity nitrate transporter 2 family genes and their responses to stress
Source: Front Plant Sci. 2023 Apr 5;14:1170048. doi: 10.3389/fpls.2023.1170048 (PMC10113457; doi:10.3389/fpls.2023.1170048)

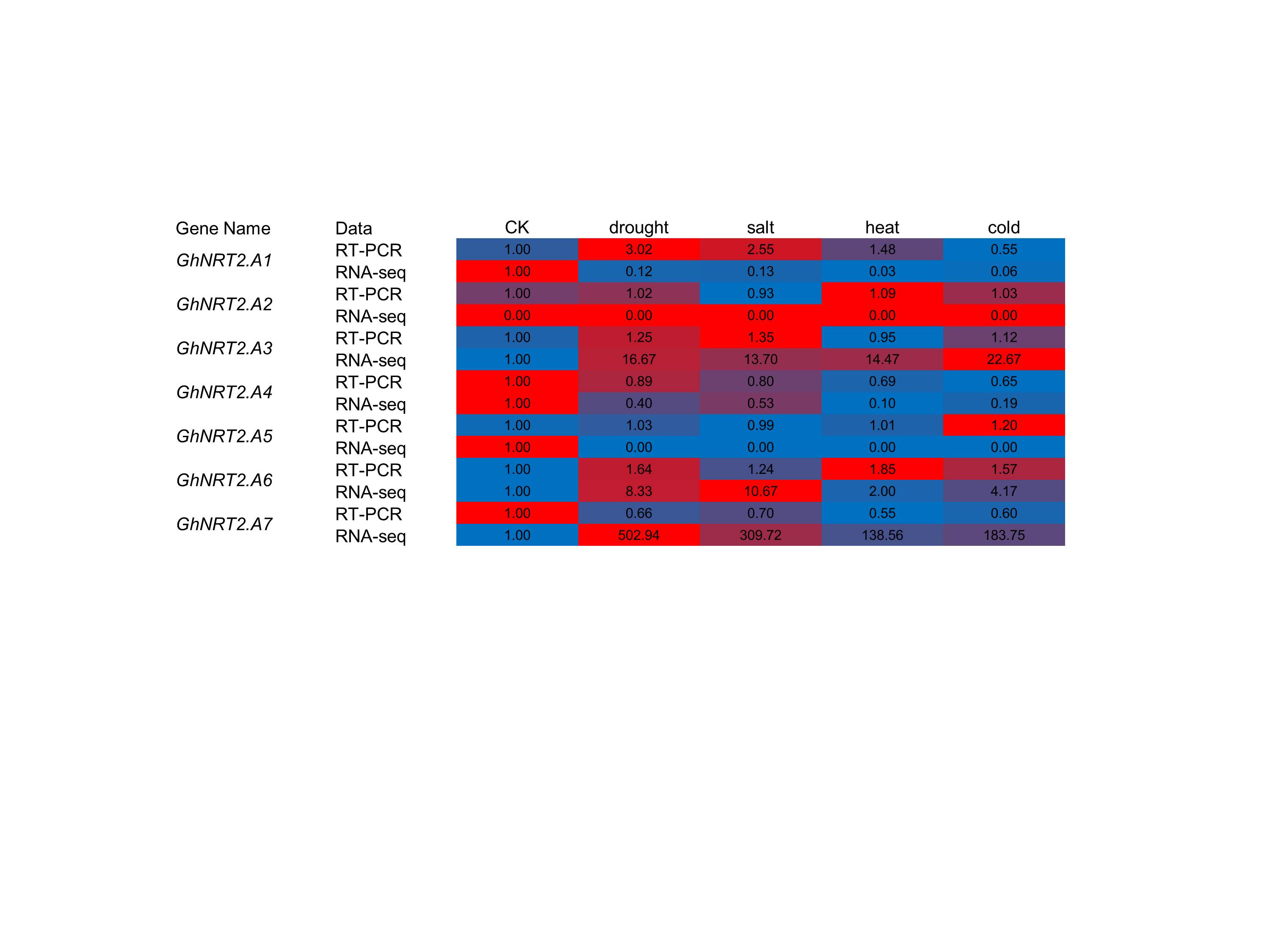

Supplement: Supplementary Figure 1 — The expression of GhNRT2.A1-A7 in different organs compared by RNA-seq data and RT-PCR data (For easy comparison, all root data were set to 1). [file Image_1.jpg]

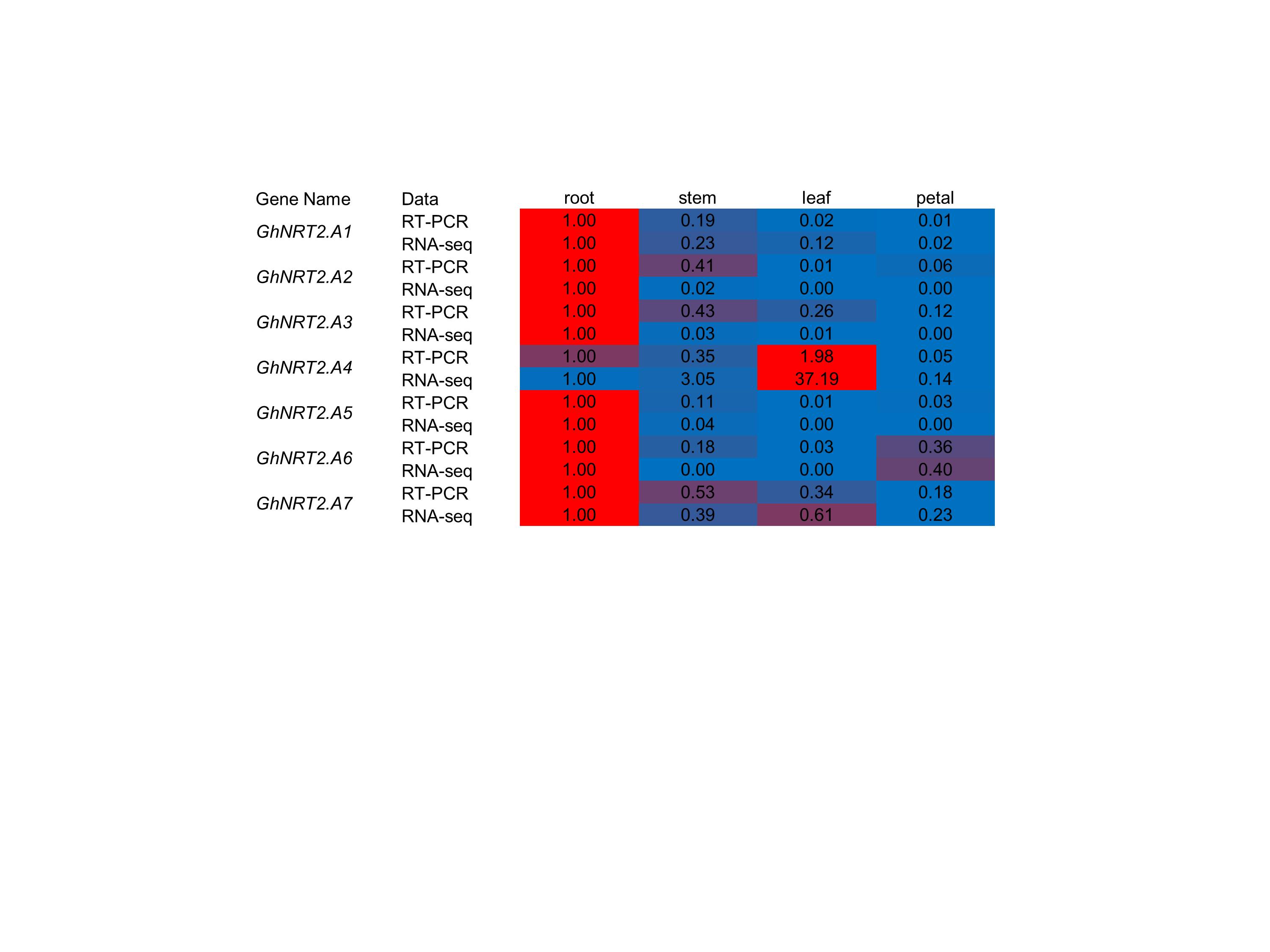

Supplement: Supplementary Figure 2 — The expression of GhNRT2.A1-A7 in different treatments compared by RNA-seq data and RT-PCR data (For easy comparison, all CD data were set to 1). [file Image_2.jpg]
